# Changes in Microbiome in Patients with Kidney Injury after Allogeneic Hematopoietic Stem Cell Transplantation

**DOI:** 10.34067/KID.0000000627

**Published:** 2024-10-24

**Authors:** Matthew H. Abramson, Insara Jaffer Sathick, Andrea Knezevic, Miguel-Angel Perales, Edgar A. Jaimes

**Affiliations:** 1 Renal Service, Memorial Sloan Kettering Cancer Center, New York, New York; 2 Icahn School of Medicine at Mount Sinai, New York, New York; 3 Weill Cornell Medical College, New York, New York; 4 Department of Epidemiology and Biostatistics, Memorial Sloan Kettering Cancer Center, New York, New York; 5 Adult Bone Marrow Transplant Service, Memorial Sloan Kettering Cancer Center, New York, New York

**Keywords:** AKI, cancer, clinical nephrology

## Abstract

**Key Points:**

- Changes in microbiome diversity index are common in patients with stem cell transplant.
- Changes in microbiome diversity do not explain the high incidence of AKI in patients with stem cell transplant.

**Background:**

AKI is a common complication of allogeneic hematopoietic cell transplantation (allo-HCT) that increases the risk of mortality. By contrast, higher diversity of intestinal microbiota at the time of neutrophil engraftment has been associated with lower mortality. We aimed to better understand kidney outcomes in relation to changes in gut diversity in this patient population, hypothesizing that patients with lower microbiome diversity at baseline and at engraftment were at higher risk of developing kidney complications.

**Methods:**

We performed a single-center retrospective study of 419 hematopoietic cell transplant recipients from 2014 to 2017 at our institution whose gut microbiota were analyzed. We defined AKI and CKD on the basis of Kidney Disease Improving Global Outcomes criteria and eGFR using the CKD Epidemiology Collaboration equation. We defined gut microbiome diversity using Shannon and Simpson reciprocal diversity indices, with higher levels indicating more diverse microbiota.

**Results:**

Simpson reciprocal diversity index and Shannon diversity index were 21.8 (interquartile range [IQR], 13.7–35.2; range, 1.6–102.5) and 3.7 (IQR, 3.2–4.2; range, 0.7–5.2) in our cohort at baseline and 6.3 (IQR, 3.7–10.4) and 2.3 (IQR, 1.7–2.8) at periengraftment, respectively. Of the 419, 263 patients (63%) developed any grade AKI in 100 days after hematopoietic cell transplantation and 114 (27%) developed grade 2+ AKI. There were no significant differences in microbiome diversity at baseline or periengraftment in patients who developed post-transplant AKI or CKD, respectively, in comparison with those who did not develop kidney complications.

**Conclusions:**

Our findings do not support the existence of a link between baseline or periengraftment gut diversity and the risk of development of AKI or CKD in patients undergoing allo-HCT. This study highlights the complex and multifactorial etiology of AKI in allo-HCT recipients and the need for additional prospective and mechanistic studies.

## Visual Abstract

**Figure:**
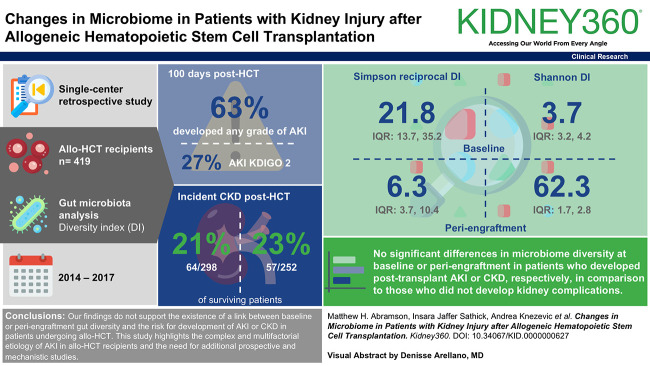

## Introduction

Allogeneic hematopoietic cell transplantation (allo-HCT) is a potential curative treatment of hematologic malignancies but is associated with high mortality because of complications, such as infections; graft-versus-host disease (GVHD); and other organ dysfunctions, including AKI.^1^ The relationship between the composition of the human microbiome and clinical outcomes after hematopoietic cell transplantation (HCT) has come under examination because of recent advances in our understanding of the microbiota. A recent large international multicenter study comprising of 1362 patients demonstrated that changes in the gut microbiota during allo-HCT were characterized by loss of diversity and domination by single taxa, and higher diversity of intestinal microbiota at the time of neutrophil engraftment was associated with lower mortality.^2^ Loss of diversity and domination by specific bacterial taxa have also been similarly demonstrated after autologous HCT, where higher intestinal diversity in the periengraftment period was associated with lower mortality, further supporting the theory that microbiome diversity plays a vital role in HCT patient outcomes.^3^ Conversely, Gu *et al.* found that higher periengraftment diversity conferred better overall outcomes.^4^ Medication exposures, including both antibiotics and nonantibiotics (*e*.*g*., emetics and opiates), have been also shown to significantly affect microbiome changes and outcomes.^5^

Dysbiosis, the pathophysiological alteration in the intestinal microbiota, could be a potential modulating factor for AKI, and abnormal kidney function in turn may affect the gut microbiome.^6^ In our previous study evaluating AKI and incident CKD in a cohort of allo-HCT recipients,^1^ we found that AKI was ubiquitous in this patient population, having occurred in 66% (403/616) of patients during their allo-HCT hospitalization, 30% (183/616) of whom developed severe AKI (stages 2–3), and 3.4% (21/616) required KRT. Furthermore, we found a strong association between AKI and nonrelapse mortality (hazard ratio, 2.77; 95% confidence interval, 1.8 to 4.27; *P* < 0.001).

In previous studies of patients after allo-HCT, AKI and loss of gut microbiome diversity was independently associated with higher mortality. Owing to rapid microbiome diversity changes and the high incidence of AKI and CKD in this high-risk population, we determined this was an ideal cohort to examine a potential association between kidney injury and microbiome diversity changes. We therefore aimed to determine whether gut microbiome biodiversity changes in patients after allo-HCT were a risk factor of developing AKI or CKD. We hypothesized that a lower baseline microbiome diversity would confer higher risk of AKI, and a lower microbiome diversity at engraftment and at the time of AKI would confer a higher risk of development of CKD.

## Methods

We retrospectively reviewed all allo-HCT performed from 2014 to 2017 at Memorial Sloan Kettering Cancer Center after obtaining Institutional Review Board approval. Patients were selected regardless of their cancer diagnosis, conditioning regimen, or GVHD prophylaxis and were thus a heterogeneous group, discussed more extensively in our prior publication.^1^ To summarize, patients received myeloablative, reduced intensity, or nonmyeloablative conditioning, depending on specific patient characteristics, per provider preference. Myeloablative regimens typically included high-dose total-body irradiation (TBI) or chemotherapy. Reduced intensity conditioning was used in patients with lower risk of GVHD and higher risk of nonrelapse mortality and typically used chemotherapy and low-dose TBI. Nonmyeloablative conditioning was typically reserved for those with the highest risk of nonrelapse mortality and included low-dose TBI (200 cGy) and chemotherapy.^7^ Transplants were considered either modified or unmodified, depending on whether the transplant was T-cell depleted. Choice of GVHD prophylaxis was also decided per institutional protocol on the basis of specific patient and disease characteristics, using either cyclophosphamide, tacrolimus, tacrolimus/sirolimus,^8^ or T-cell depletion (which did not require pharmacologic immunosuppression).^9^

Patients with microbiome data from stool specimens collected routinely as part of their transplant care were included in the study. Shannon and Simpson reciprocal diversity indices (DIs) were calculated for all samples.^2,10^ Higher DIs indicate a higher microbiome diversity. Alpha-diversity is a mathematical value that summarizes a microbial community according to the count of unique species and how evenly their frequencies are distributed. The higher the number of unique species and the more evenly they are distributed in the community, the higher the alpha-diversity. We calculated the alpha-diversity index using the inverse Simpson index and the alternative method for alpha-diversity, the Shannon index. These two metrics are highly correlated with one another, but the Simpson index is slightly less sensitive to the long tail of rare bacteria than the Shannon index. Baseline DI was defined as the closest DI within 7 days of conditioning (day −7 to day +7). AKI was measured as a binary and time-to-event variable, restricted to 100 days of follow-up after HCT. Patients were recorded as having any grade AKI or grade 2+ (severe) AKI. Death before development of AKI was considered a competing event in time-to-event analyses.

We categorized AKI by stage, using Kidney Disease Improving Global Outcomes criteria,^11,12^ as follows: Stage 1 (creatinine rise of ≥0.3 mg/dl or 1.5× baseline), Stage 2 (creatinine rise of at least 2× baseline), and Stage 3 (creatinine rise of least 3× baseline). All patients who required ≥1 session of KRT, either by intermittent hemodialysis or by continuous KRT, were considered as Stage 3. We defined severe AKI as AKI Stage 2–3, that is, at least doubling of serum creatinine from baseline. Baseline serum creatinine was defined as creatinine level on admission for HCT, before any conditioning, which occurred around 11 days (interquartile range [IQR], −15 to −10) before transplant. Baseline eGFR was calculated using the CKD Epidemiology Collaboration formula.^13^ Baseline CKD was defined as admission eGFR <60 ml/min per 1.73 m^2^. Death before the development of AKI was considered a competing event in time-to-event analyses. In binary analysis, deaths before AKI were excluded.

To assess the association between baseline DI and the subsequent development of any grade AKI or grade 2+ AKI, baseline DI variables were compared between groups using Wilcoxon rank-sum test and in a competing-risks Cox proportional hazards regression model with baseline DI as a covariate. In a separate model, longitudinal DI measures were used as a time-dependent covariate in competing-risks Cox proportional hazards regression. Any available longitudinal DI measures were used after date of HCT up to 100 days after HCT and any DI measured after development of AKI was excluded. Because DI measures were not collected systematically for all patients over time, it was difficult to discern trends and make comparisons between groups. If a patient had multiple measures in 1 week, the first one was used, and any DI measured after development of AKI was excluded. We defined periengraftment DI as the median diversity index from day +7 through day +21 of HCT.

Incident CKD was defined as eGFR <60 cc/min at 6 and 12 months after HCT in patients who did not have baseline CKD. Periengraftment DI was compared between patients with CKD and normal kidney function at 6 and 12 months. Stage of CKD was defined as 3a (eGFR 45–59.9 cc/min), 3b (30–44.9 cc/min), 4 (15–29.9 cc/min), and 5 (<15 cc/min).

Peri-AKI DI was defined as the closest DI within 7 days of AKI onset. For the subset of patients who developed at least grade 1 AKI, the peri-AKI DI was compared by subsequent development of CKD. Periengraftment and peri-AKI DI were compared between CKD and normal kidney function groups with the Wilcoxon rank-sum test. For patients who developed grade 3 AKI, the peri-AKI DI was compared by subsequent need for KRT.

## Results

### Patient Characteristics

A total of 616 patients underwent an allo-HCT during the study period. 491 patients had their microbiota analyzed, and 419 of those had a baseline DI. As such, we limited our analysis to the 419 patients with baseline DI. The median age was 57 years (range, 19–78), and 165 (39%) were female (Table 1). Most of the cohort (329/419 or 79%) was White, 25 of 419 (6%) Black, and 23 of 419 (5%) Hispanic. Acute leukemia was the predominant indication for allo-HCT, comprising of 210 patients (50%), followed by non-Hodgkin lymphoma in 16% (67/419), myelodysplastic syndrome in 16% (62/419), and multiple myeloma in 11% (44/419), with the rest comprising of myeloproliferative disorders, Hodgkin disease, and other nonmalignant disorders. The median HCT-specific comorbidity index was ≥3 in 201 patients (48%), indicating a higher risk cohort.^14^ In terms of GVHD prophylaxis, 44% (184/419) of patients received T-cell–depleted transplants and 56% (206/419) received calcineurin inhibitor–based regimens. One of four patients had CKD at baseline, and none of the patients had a history of solitary kidney or kidney transplant.

**Table 1:** Baseline patient and hematopoietic cell transplantation treatment characteristics (*N*=419)

Demographics	
Age, yr, median (range)	57 (19–78)
**Sex**	
Female	165 (39%)
BMI, median (IQR)	27.3 (24.0–30.8)
**Race/ethnicity**	
Asian, non-Hispanic	19 (5%)
Black, non-Hispanic	25 (6%)
Hispanic, any race	23 (5%)
Not reported	21 (5%)
Other	2 (<1%)
White, non-Hispanic	329 (79%)
**Disease**	
Leukemia	210 (50%)
Non-Hodgkin lymphoma	67 (16%)
Myelodysplastic syndrome	62 (16%)
Multiple myeloma	44 (11%)
Myeloproliferative disorder	17 (4%)
Hodgkin disease	10 (2%)
Nonmalignant disorders	9 (2%)
Baseline albumin, g/dl, median (IQR)	3.5 (3.3–3.8)
Baseline creatinine, mg/dl, median (IQR)	0.8 (0.7–1.0)
Baseline eGFR, ml/min, median (IQR)	94 (80–119)
Baseline eGFR <60 ml/min	24 (6%)
**HCT-CI**	
0	73 (17%)
1–2	145 (35%)
3+	201 (48%)
Baseline Simpson reciprocal diversity index, median (IQR)	21. 8 (13.7–35.2)
Baseline Shannon diversity index, median (IQR)	3.7 (3.2–4.2)
**Treatment**	
Conditioning intensity	
*Myeloablative*	265 (63%)
*Nonmyeloablative*	32 (8%)
*Reduced intensity*	122 (29%)
Conditioning regimen	
*Chemotherapy-based*	309 (74%)
*TBI-based with low-dose TBI (200–400 cGy)*	49 (12%)
*TBI-based with high-dose TBI (1375 cGy)*	61 (14%)
GVHD prophylaxis	
*Ex vivo T-cell depletion*	184 (44%)
*CNI-based*	235 (56%)
HLA	
*Unrelated identical*	227 (54%)
*Related identical sibling*	117 (28%)
*Unrelated nonidentical*	41 (10%)
*Related haploidentical*	33 (8%)
*Related nonidentical*	1 (<1%)
Patient CMV positive	237 (57%)
Donor CMV positive	200 (48%)

BMI, body mass index; cGy, centiGray; CMV, cytomegalovirus; CNI, calcineurin inhibitor; GVHD, graft-versus-host disease; HCT-CI, hematopoietic cell transplantation–specific comorbidity index; HLA, human leukocyte antigens; IQR, interquartile range; TBI, total body irradiation; TCD, T-cell–depleted transplant.

### AKI and CKD Analyses

**Figure 1:**
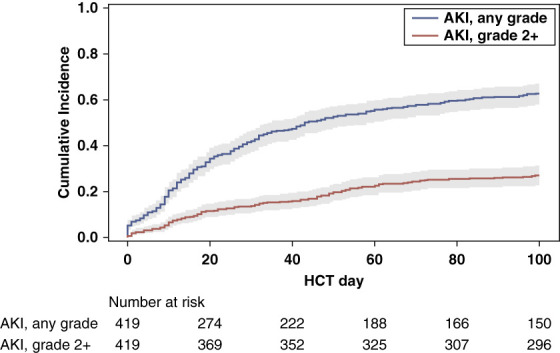
**Cumulative incidence of any grade AKI and grade 2+ AKI in 100 days of follow-up after HCT**. At 100 days after HCT, the cumulative incidence of any grade AKI is 68% (95% CI, 58 to 67) and the cumulative incidence of grade 2+ AKI is 27% (95% CI, 23 to 32). CI, confidence interval; HCT, hematopoietic cell transplantation.

In this cohort, 263 of 419 (63%) developed any grade AKI within the first 100 days after HCT and 6 patients (1%) died without developing AKI. Grade 2+ AKI was observed in 114 patients (27%) (Figure 1).

Of the 419 patients with baseline DI, 330 had available periengraftment DIs, which were used for this analysis. Patients with CKD at baseline (19 patients) and six patients with missing follow-up eGFR values were excluded from this analysis. Incident CKD at 6 months post-HCT was observed in 64 of 298 of surviving patients (21%) and CKD at 12 months in 57 of 252 (23%); the remainder of patients either had normal kidney function or died before the time point.

### Changes in DI Over Time

DI was first assessed at baseline, defined as the DI closest to the start of HCT conditioning, within 14 days (day −7 to day +7) of conditioning. The median Simpson reciprocal DI at baseline was 21.8 (IQR, 13.7–35.2; range, 1.6–102.5), and the median Shannon DI was 3.7 (IQR, 3.2–4.2; range, 0.7–5.2). The median baseline DI was 1 day after the start of conditioning and 6 days before HCT.

DI was next assessed in the peri-engraftment period. Periengraftment DI was defined as the median DI value from days 7 to 21 (day +7 to +21) after allo-HCT. Values were available for 330 patients. The median Simpson reciprocal DI at periengraftment was 6.4 (IQR, 3.9–10.3; range,1.7–41.4), and the median Shannon DI was 2.3 (IQR, 1.7–2.8; range, 0.6–4.1).

**Figure 2:**
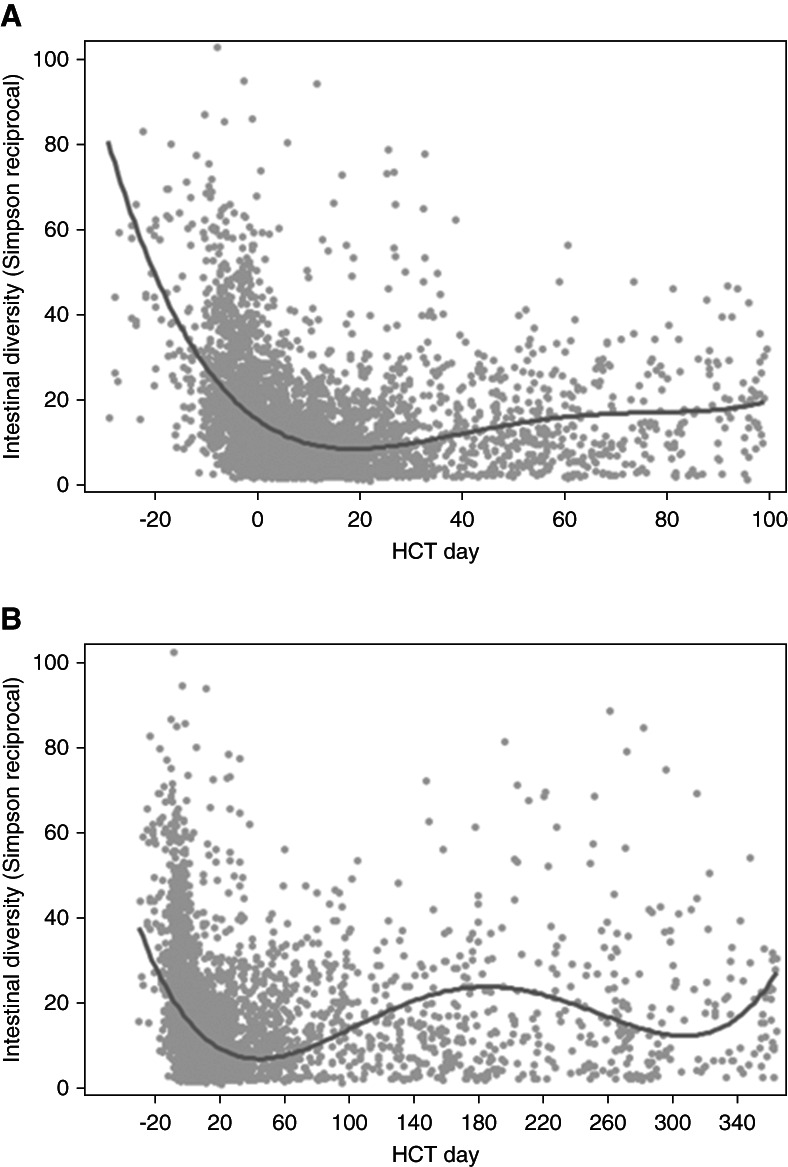
**Graphical depiction of diversity trends over the first 100 days and through the first year.** (A) Change in diversity of intestinal microbiota 100 days after HCT. (B) Change in diversity of intestinal microbiota 1 year after HCT. The trend lines are fourth-order polynomial lines of best fit, meant to capture up to three trend changes in DI over time. DI, diversity index.

Graphical depiction of diversity trends over the first 100 days and through the first year is shown in Figure 2, A and B, respectively. Diversity trends were similar between those who received T-cell–depleted versus calcineurin inhibitor-based GVHD prophylaxis, in either absence or presence of AKI (Supplemental Figure 4).

### Dynamic Landmark Analyses

#### Baseline DI did not Affect AKI Development

**Figure 3:**
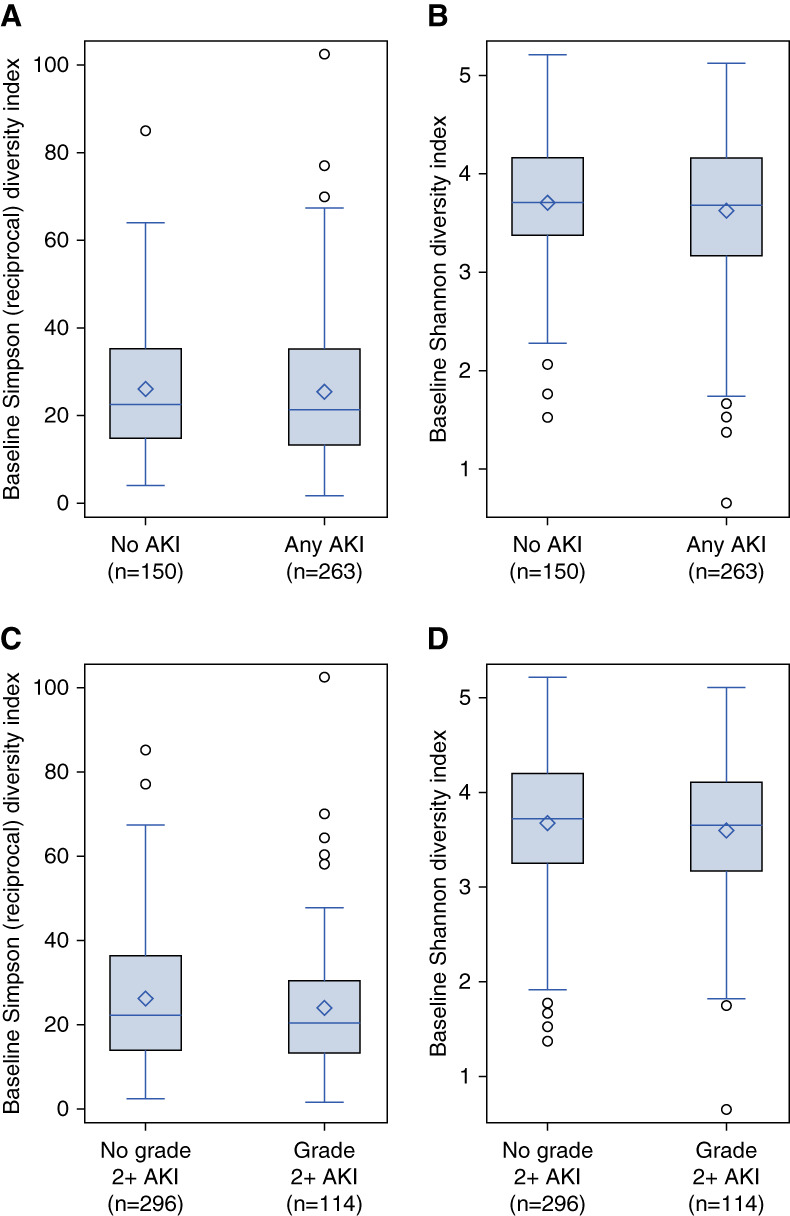
**Microbiome diversity indices and subsequent development of AKI.** (A) Simpson reciprocal index at baseline by any AKI development in 100 days after HCT showed no association between the development of AKI and this index (*P* = 0.42). (B) Shannon diversity index at baseline by any AKI development in 100 days after HCT showed no association between the development of AKI and this index (*P* = 0.49). (C) Simpson reciprocal diversity index at baseline by grade 2+ AKI development in 100 days after HCT showed no association between the development of AKI and this index (*P* = 0.18). (D) Shannon diversity index at baseline by grade 2+ AKI development in 100 days after HCT showed no association between the development of severe (grade 2+) AKI and the Shannon diversity index (*P* = 0.23). Six patients with death and no AKI were excluded from (A) and (B), and nine patients with death and no grade 2+ AKI were excluded from (C) and (D).

A total of 419 patients had baseline DI available. Baseline DI was similar for patients who developed any AKI versus those who did not (Figure 3, A and B) and similar for patients who developed grade 2+ AKI versus those who did not (Figure 3, C and D).

**Figure 4:**
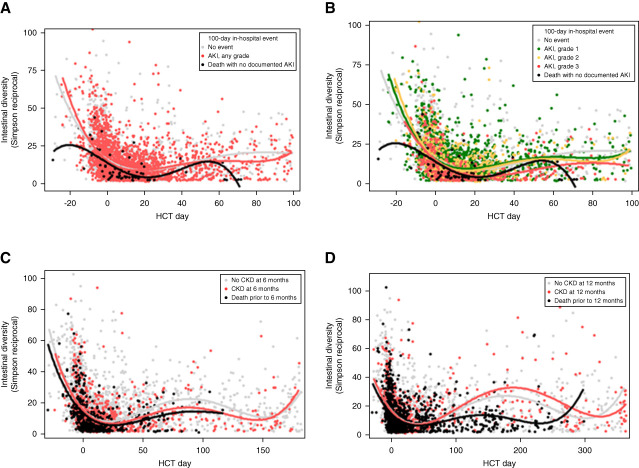
**Change in diversity of intestinal microbiota during the HCT period.** (A) Stratified by AKI status. (B) Stratified by AKI severity. (C) Trends in diversity index by CKD at 6 months (shown over 6 months after HCT). (D) Stratified by CKD status at 12 months. The trend lines are fourth-order polynomial lines of best fit, meant to capture up to three trend changes in DI over time.

Figure 4A compares the 100-day DI trend in patients without AKI and with AKI and Figure 4B by each AKI grade. As shown in the univariate analysis (Table 2) and multivariate analysis (Supplemental Table 3), changes in Simpson reciprocal DI or Shannon DI did not alter AKI outcomes.

**Table 2:** Results from competing-risks Cox proportional hazards regression

DI	Any Grade AKI		Grade 2+ AKI	
	HR (95% CI)	*P* Value	HR (95% CI)	*P* Value
**Simpson reciprocal DI**				
HR for every ten-unit increase				
*Baseline*	0.98 (0.91 to 1.06)	0.67	0.91 (0.80 to 1.04)	0.18
*Time-dependent covariate*	1.01 (0.90 to 1.14)	0.88	0.90 (0.75 to 1.07)	0.22
*Periengraftment^a^*	1.21 (0.93 to 1.55)	0.16	0.81 (0.52 to 1.27)	0.35
**Shannon DI**				
HR for every one-unit increase				
*Baseline*	0.93 (0.78 to 1.10)	0.39	0.85 (0.66 to 1.10)	0.22
*Time-dependent covariate*	1.07 (0.93 to 1.22)	0.36	0.91 (0.74 to 1.10)	0.32
*Periengraftment^a^*	1.25 (1.00 to 1.56)	0.052	0.85 (0.63 to 1.14)	0.28

Results from competing-risks Cox proportionalhazards regression using baseline diversity index values do not show a significant association with development of any grade AKI or grade 2+ AKI (Table 2). Longitudinal diversity index measures used as a time-dependent covariate are also not significantly associated with development of any grade AKI or grade 2+ AKI.

Models for diversity index at baseline and diversity index as a time-dependent covariate use 419 patients. Diversity index values between day 0 and 100 (or development of AKI) are used for the time-dependent covariate. For any grade AKI time-dependent covariate models, 3116 diversity index observations are used (median five per patient) and for grade 2+ AKI time-dependent covariate models, 3609 diversity index observations are used (median seven per patient).

Patients with a periengraftment diversity index available who did not experience an event before day 21 were included in a landmark time-to-event analysis starting at day 21 for any grade AKI (*n*=268 with 123 events and five deaths) or grade 2+ AKI (*n*=354 with 64 events and nine deaths) using periengraftment diversity index as a covariate.

There were no significant results, adjusting for the following covariates: patient ethnicity, baseline albumin, conditioning intensity, conditioning regimen, and graft-versus-host disease prophylaxis group. See Supplemental Table 3 for this multivariate analysis. CI, confidence interval; DI, diversity index; HR, hazard ratio.

a Landmark analysis starting at day 21 after hematopoietic cell transplantation.

#### Periengraftment DI Did Not Affect Development of CKD

**Figure 5:**
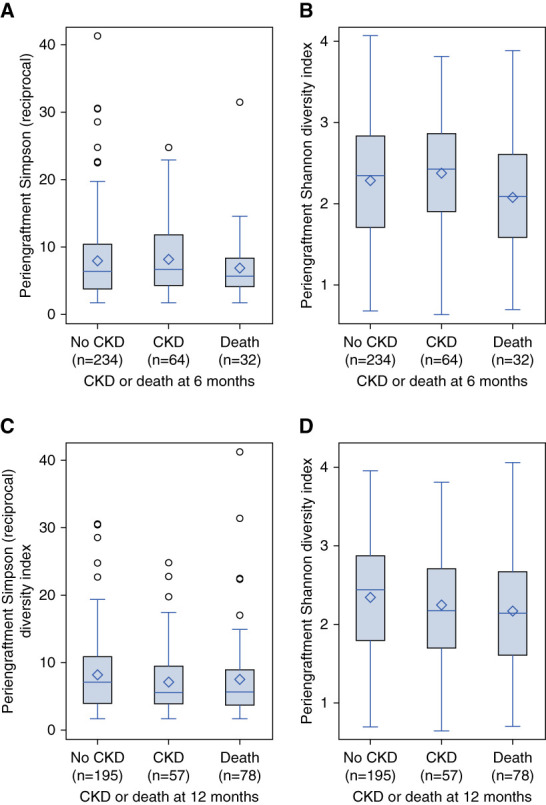
**Periengraftment diversity and development of CKD.** (A) Periengraftment Simpson reciprocal diversity index by CKD at 6 months after HCT. There was no association found between the development of CKD at 6 months and the reciprocal Simpson periengraftment diversity index (*P* = 0.38). (B) Periengraftment Shannon diversity index by CKD at 6 months after HCT. There was no association found between the development of CKD at 6 months and the Shannon periengraftment diversity index (*P* = 0.43). (C) Periengraftment Simpson reciprocal diversity index by CKD at 12 months after HCT. There was no association found between the development of CKD at 12 months and the reciprocal Simpson periengraftment diversity index (*P* = 0.27). (D) Periengraftment Shannon diversity index by CKD at 12 months after HCT. There was no association found between the development of CKD at 12 months and the Shannon periengraftment diversity index (*P* = 0.17).

Figure 4C compares the DI trend in patients who developed CKD at 6 months and Figure 4D at 12 months. Values of periengraftment DI were similar for patients who developed CKD versus those who did not, at both 6 months (Figure 5, A and B) and 12 months (Figure 5, C and D).

#### DI at the Time of AKI (Peri-AKI) Did Not Increase Risk of CKD

**Figure 6:**
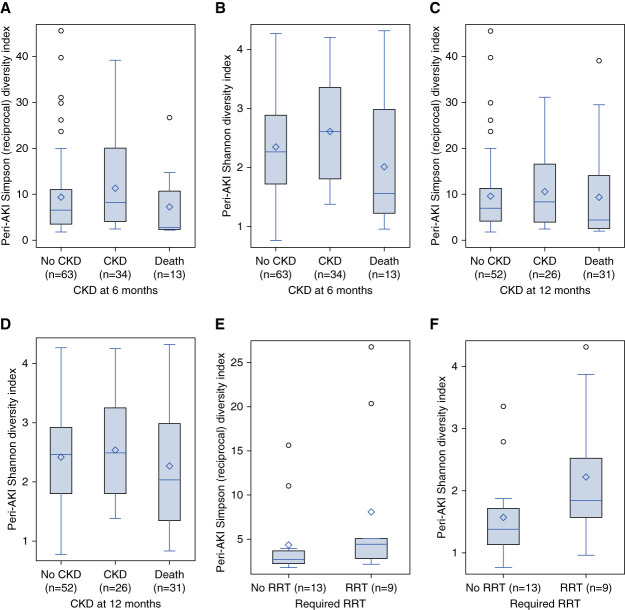
**Peri-AKI diversity index and CKD.** (A) Peri-AKI reciprocal Simpson diversity index and subsequent development of CKD at 6 months. There was no association found between the development of CKD or death at 6 months and reciprocal Simpson diversity index (*P* = 0.24) at the time of AKI. Peri-AKI is the closest diversity index to date of AKI onset. (B) Peri-AKI Shannon diversity index and subsequent development of CKD at 6 months. There was no association found between the development of CKD or death at 6 months and the Shannon diversity index (*P* = 0.19) at the time of AKI. Peri-AKI is the closest diversity index to date of AKI onset. (C) Peri-AKI reciprocal Simpson diversity index and subsequent development of CKD at 12 months. There was no association found between the development of CKD or death at 12 months and the reciprocal Simpson diversity index (*P* = 0.61) at the time of AKI. Peri-AKI is the closest diversity index to the date of AKI onset. (D) Peri-AKI Shannon diversity index and subsequent development of CKD. There was no association found between the development of CKD or death at 12 months and the Shannon diversity index (*P* = 0.72) at the time of AKI. Peri-AKI is the closest diversity index to the date of AKI onset. (E) Grade 3 peri-AKI Simpson reciprocal diversity index and subsequent need for RRT. There was no association found between the need for RRT and Simpson reciprocal diversity index (*P* = 0.10) at the time of AKI onset. Peri-AKI is the closest diversity index to the date of AKI onset. (F) Grade 3 peri-AKI Shannon diversity index and subsequent need for RRT. There was no association found between the need for RRT and Shannon diversity index (*P* = 0.12) at the time of AKI onset. Peri-AKI is the closest diversity index to the date of AKI onset. RRT, renal replacement therapy;

Peri-AKI DI was defined as the DI value closest to AKI onset (within 7 days). For the subset of patients who developed at least grade 1 AKI (*N*=263), peri-AKI DI values were available for 111. We found that peri-AKI DI did not increase risk of development of CKD at either 6 months (Figure 6, A and B) or 12 months (Figure 6, C and D).

#### Grade 3 Peri-AKI DI and Subsequent Need for RRT

For the subset of patients who developed grade 3 AKI (*N*=40), peri-AKI DI values were available for 22 patients. Nine patients required KRT after HCT. Peri-AKI DI values trended higher in KRT patients (median Simpson reciprocal 4.5 versus 2.7; median Shannon 1.8 versus 1.4), but the group difference was not statistically significant (Simpson *P* = 0.10; Shannon *P* = 0.12) (Figure 6, E and F).

## Discussion

We have previously demonstrated a high incidence of AKI in 66% of patients undergoing an allogeneic hematopoietic stem cell transplantation, with stage 3 AKI occurring in 10% of patients and 3% requiring renal replacement therapy.^1^ Given the higher mortality associated with AKI (hazard ratio, 2.77) and emerging evidence for the effect of gut diversity on patient outcomes, we investigated the association between changes in gut microbiome and the development of AKI and CKD in this patient population. While we hypothesized that baseline diversity would affect development of AKI and diversity at the time of AKI and engraftment would confer risk of CKD, our study was unable to demonstrate any of these associations.

The effect of gut microbiome disruption as a cause or consequence of chronic diseases is currently an area of scientific interest.^15–17^ The interaction between the gut microbiome and the kidney through the colorenal axis is being recognized as a modulating factor for AKI.^6^ In a recent experimental animal model study, colonizing germ-free mice with post-AKI microbiota was shown to worsen ischemia–reperfusion injury severity in recipient mice compared with colonizing with microbiota from sham operated mice.^18^ A bidirectional relationship between the kidney and the intestine has been postulated, indicating that intestinal dysbiosis, inflammation, and leaky gut may not only be consequences, but also important determinants of the severity of AKI. Short-chain fatty acids (SCFAs), which are the end products of fermentation of dietary fibers produced by gut microbiota, may be potent mediators of this interaction. When released into systemic circulation, SCFAs have effects on the juxtaglomerular apparatus, reducing renin release, subsequently leading to reduction in BP.^19^ SCFAs also modulate cell signal transduction processes using G-protein coupled receptors and may be involved in the regulation of immune function, as well as the autophagy pathway.^20–24^ Alterations in microbiome and administration of SCFAs have been shown to mitigate severity of inflammation and AKI in mouse models of kidney injury.^23,25^

In our study, we found that changes in microbiome diversity were not linked to the development of AKI or CKD in patients undergoing allo-HCT. There were similar changes in microbiome diversity in all groups at both day +100 and after 1 year. Diversity at the time of stem cell conditioning did not predict development or severity of AKI. Furthermore, neither periengraftment diversity nor diversity at AKI onset was found to be a risk factor of developing CKD at either 6 or 12 months. Similarly, CKD development was unaffected by microbiome diversity at the time of AKI onset. Diversity at the time of AKI did not increase the risk of requiring KRT.

Our study did not demonstrate a direct association between detrimental changes in gut biodiversity with the development of AKI or CKD. There may be several reasons for the lack of association. First, AKI after allo-HCT is often multifactorial without an evident single major etiologic factor. Second, the same factors affecting kidney function may also affect the gut microbiome; for example, antibiotics used for prophylaxis during the early phase after HCT may also affect the gut diversity. For instance, antibiotics, including vancomycin, meropenem, and gentamicin, have been associated with higher prevalence of pathological bacteria, including *Enterobacteriaceae*, and reduction in *Bifidobacterium* species, ^26^ and other studies have shown reduction in biodiversity and overgrowth of *Clostridium difficile*.^27^

Our study was retrospective, and it may not have been sufficiently powered to demonstrate a direct association between gut microbiome diversity and kidney outcomes. Furthermore, our dataset was limited in that it did not include specific microbiome compositions, and thus, we were not able to associate different taxa with kidney outcomes. Other potential limitations include the effect of conditioning on the baseline DI in some patients whose baseline DI was measured after the initiation of conditioning. Furthermore, only 419 of 616 patients had a baseline DI available, and thus, 197 patients were excluded from the study. Supplemental Figure 2 and Supplemental Table 1 compare the included and excluded cohorts, and the excluded cohorts had higher rates of AKI and severe AKI, perhaps suggesting that some of the sicker patients were excluded from our study. In addition, our cohort was heterogeneous in many respects, especially in regard to different cancer types, conditioning, and GVHD prophylaxis regimens. We reviewed the subcohorts of GVHD prophylaxis, T-cell depletion, and calcineurin inhibitor-based regimens and found the trends of diversity over time were not different, and thus, did not feel further subgroup assessment was indicated. Despite these limitations, the study concept is novel and first of its kind exploring the gut–kidney axis in human patients in a real-world study. Changes in antibiotic choice, conditioning, or fecal transplant are interventions that could be explored with the goal to improve long-term kidney outcomes and reduce nonrelapse mortality. Future studies may be able to assess for a potential link between microbiome changes and the association with kidney injury.

## Supplementary Material

## Data Availability

All data are included in the manuscript and/or supporting information.

## Supplemental Material

Supplemental Table 1. Baseline patient and HCT treatment characteristics by study inclusion.

Supplemental Figure 2. Cumulative incidence of any grade AKI and grade 2+ AKI in 100 days of follow-up after HCT, by study inclusion.

Supplemental Table 3. Results from competing-risks Cox proportional hazards regression (adjusted analysis).

Supplemental Figure 4. Changes in diversity index over time comparing T-cell depletion and CNI-based GVHD prophylaxis and stratified by AKI status.
