# Supplementary material for: Changes in Microbiome in Patients with Kidney Injury after Allogeneic Hematopoietic Stem Cell Transplantation
Source: Kidney360. 2024 Oct 24;6(1):58–68. doi: 10.34067/KID.0000000627 (PMC11793185; doi:10.34067/KID.0000000627)
Supplement: Supplementary file 1 [file kidney360-6-058-s001.pdf]

ASN American Society of Nephrology
JASN Journal of the American Society of Nephrology
KIDNEY360 Accessing Our World From Every Angle
CJASN Clinical Journal of the American Society of Nephrology

ASN Journal Disclosure Form

As per ASN journal policy, I have disclosed any financial relationships or commitments I have held in the past 36 months as included below. I have listed my Current Employer below to indicate there is a relationship requiring disclosure. If no relationship exists, my Current Employer is not listed.

M. Abramson reports the following:
Employer: Icahn School of Medicine at Mount Sinai

I understand that the information above will be published within the journal article, if accepted, and that failure to comply and/or to accurately and completely report the potential financial conflicts of interest could lead to the following: 1) Prior to publication, article rejection, or 2) Post-publication, sanctions ranging from, but not limited to, issuing a correction, reporting the inaccurate information to the authors' institution, banning authors from submitting work to ASN journals for varying lengths of time, and/or retraction of the published work.

Name: Matthew Abramson
Manuscript ID: K360-2024-000288R1
Manuscript Title: Changes in microbiome in patients with kidney injury after allogeneic hematopoietic stem cell transplantation
Date of Completion: July 31, 2024
Disclosure Updated Date: July 31, 2024

ASN
American Society of Nephrology
JASN
Journal of the American Society of Nephrology
KIDNEY360
Accessing Our World From Every Angle
CJASN
Clinical Journal of the American Society of Nephrology

ASN Journal Disclosure Form

As per ASN journal policy, I have disclosed any financial relationships or commitments I have held in the past 36 months as included below. I have listed my Current Employer below to indicate there is a relationship requiring disclosure. If no relationship exists, my Current Employer is not listed.

I. Jaffer Sathick reports the following:
Employer: Memorial Sloan Kettering Cancer Center

I understand that the information above will be published within the journal article, if accepted, and that failure to comply and/or to accurately and completely report the potential financial conflicts of interest could lead to the following: 1) Prior to publication, article rejection, or 2) Post-publication, sanctions ranging from, but not limited to, issuing a correction, reporting the inaccurate information to the authors' institution, banning authors from submitting work to ASN journals for varying lengths of time, and/or retraction of the published work.

Name: Insara Jaffer Sathick
Manuscript ID: K360-2024-000288R1
Manuscript Title: Changes in microbiome in patients with kidney injury after allogeneic hematopoietic stem cell transplantation
Date of Completion: July 31, 2024
Disclosure Updated Date: July 31, 2024

ASN American Society of Nephrology | JASN Journal of the American Society of Nephrology | KIDNEY360 Accessing Our World From Every Angle | CJASN Clinical Journal of the American Society of Nephrology

ASN Journal Disclosure Form

As per ASN journal policy, I have disclosed any financial relationships or commitments I have held in the past 36 months as included below. I have listed my Current Employer below to indicate there is a relationship requiring disclosure. If no relationship exists, my Current Employer is not listed.

E. Jaimes reports the following:
Employer: Memorial Sloan Kettering Cancer Center

I understand that the information above will be published within the journal article, if accepted, and that failure to comply and/or to accurately and completely report the potential financial conflicts of interest could lead to the following: 1) Prior to publication, article rejection, or 2) Post-publication, sanctions ranging from, but not limited to, issuing a correction, reporting the inaccurate information to the authors' institution, banning authors from submitting work to ASN journals for varying lengths of time, and/or retraction of the published work.

Name: Edgar A. Jaimes
Manuscript ID: K360-2024-000288R2
Manuscript Title: Changes in microbiome in patients with kidney injury after allogeneic hematopoietic stem cell transplantation
Date of Completion: September 19, 2024
Disclosure Updated Date: February 5, 2024

ASN American Society of Nephrology | JASN Journal of the American Society of Nephrology | KIDNEY360 Accessing Our World From Every Angle | CJASN Clinical Journal of the American Society of Nephrology

ASN Journal Disclosure Form

As per ASN journal policy, I have disclosed any financial relationships or commitments I have held in the past 36 months as included below. I have listed my Current Employer below to indicate there is a relationship requiring disclosure. If no relationship exists, my Current Employer is not listed.

A. Knezevic reports the following:
Employer: Memorial Sloan Kettering Cancer Center; and Consultancy: ByHeart, Inc.

I understand that the information above will be published within the journal article, if accepted, and that failure to comply and/or to accurately and completely report the potential financial conflicts of interest could lead to the following: 1) Prior to publication, article rejection, or 2) Post-publication, sanctions ranging from, but not limited to, issuing a correction, reporting the inaccurate information to the authors' institution, banning authors from submitting work to ASN journals for varying lengths of time, and/or retraction of the published work.

Name: Andrea Knezevic
Manuscript ID: K360-2024-000288R2
Manuscript Title: Changes in microbiome in patients with kidney injury after allogeneic hematopoietic stem cell transplantation
Date of Completion: September 5, 2024
Disclosure Updated Date: March 26, 2024

ASN American Society of Nephrology | JASN Journal of the American Society of Nephrology | KIDNEY360 Accessing Our World From Every Angle | CJASN Clinical Journal of the American Society of Nephrology

ASN Journal Disclosure Form

As per ASN journal policy, I have disclosed any financial relationships or commitments I have held in the past 36 months as included below. I have listed my Current Employer below to indicate there is a relationship requiring disclosure. If no relationship exists, my Current Employer is not listed.

M. Perales reports the following:
Employer: Memorial Sloan Kettering Cancer Center; Consultancy: Merck; Ownership Interest: Neximmune, Omeros, Orcabio; Research Funding: Institutional research support for clinical trials from Allogene, Incyte, Kite/Gilead, Miltenyi Biotec, Nektar Therapeutics, and Novartis.; Honoraria: Adicet, Allogene, AlloVir, Caribou Biosciences, Celgene, Bristol-Myers Squibb, Equilium, Exevir, ImmPACT Bio, Incyte, Karyopharm, Kite/Gilead, Merck, Miltenyi Biotec, MorphoSys, Nektar Therapeutics, Novartis, Omeros, OrcaBio, Syncopation, VectivBio AG, and Vor Biopharma; and Other Interests or Relationships: Data safety and monitoring boards for Cidara Therapeutics, Medigene, and Sellas Life Sciences.

I understand that the information above will be published within the journal article, if accepted, and that failure to comply and/or to accurately and completely report the potential financial conflicts of interest could lead to the following: 1) Prior to publication, article rejection, or 2) Post-publication, sanctions ranging from, but not limited to, issuing a correction, reporting the inaccurate information to the authors' institution, banning authors from submitting work to ASN journals for varying lengths of time, and/or retraction of the published work.

Name: Miguel-Angel Perales
Manuscript ID: K360-2024-000288R2
Manuscript Title: Changes in microbiome in patients with kidney injury after allogeneic hematopoietic stem cell transplantation
Date of Completion: October 15, 2024
Disclosure Updated Date: October 15, 2024
