# Supplementary material for: Changes in Microbiome in Patients with Kidney Injury after Allogeneic Hematopoietic Stem Cell Transplantation
Source: Kidney360. 2024 Oct 24;6(1):58–68. doi: 10.34067/KID.0000000627 (PMC11793185; doi:10.34067/KID.0000000627)
Supplement: Supplementary file 2 [file kidney360-6-058-s002.pdf]

Supplemental:

Supplemental Table 1. Baseline patient and HCT treatment characteristics by study inclusion

	Included (N=419)	Excluded – No microbiota data (N=197)	P-value
Age, years – median (range)	57 (19, 78)	60 (24, 79)	0.18
Sex			
Male	254 (61%)	110 (56%)	0.26
Female	165 (39%)	87 (44%)	
BMI – median (IQR)	27.3 (24.0, 30.8)	26.5 (15.7, 78.5)	0.37
Race/Ethnicity			0.48
White, non-Hispanic	329 (79%)	152 (77%)	
Black, non-Hispanic	25 (6%)	9 (5%)	
Hispanic, any race	23 (5%)	16 (8%)	
Asian, non-Hispanic	19 (5%)	10 (5%)	
Other	2 (<1%)	3 (2%)	
Not reported	21 (5%)	7 (4%)	
Disease			0.78
Leukemia	210 (50%)	93 (47%)	
Non-Hodgkin's Lymphoma	67 (16%)	33 (17%)	
Myelodysplastic Syndrome	62 (16%)	37 (19%)	
Multiple Myeloma	44 (11%)	17 (9%)	
Myeloproliferative Disorder	17 (4%)	7 (4%)	
Hodgkin's Disease	10 (2%)	5 (3%)	
Non-Malignant Disorders	9 (2%)	5 (3%)	
Baseline Albumin, g/dL – median (IQR)	3.5 (3.3, 3.8)	3.5 (3.3, 3.8)	0.41
Baseline Creatinine, mg/dL – median (IQR)	0.80 (0.70, 1.0)	0.80 (0.70, 0.90)	0.01
Baseline eGFR, mL/min – median (IQR)	94 (80, 119)	101 (86, 123)	0.14
Baseline eGFR <60 mL/min	24 (6%)	11 (6%)	0.99
HCT-CI			0.05
0	73 (17%)	34 (17%)	
1-2	145 (35%)	50 (25%)	
3+	201 (48%)	113 (57%)	
**Treatment**			
Conditioning Intensity			0.02
Myeloablative	265 (63%)	102 (52%)	
Non-myeloablative	32 (8%)	18 (9%)	

Reduced Intensity	122 (29%)	77 (39%)	
Conditioning regimen			0.01
Chemotherapy-based	309 (74%)	153 (78%)	
TBI-based with low dose TBI (200-400 cGy)	49 (12%)	31 (16%)	
TBI-based with high dose TBI (1375 cGy)	61 (14%)	13 (7%)	
GVHD prophylaxis			<0.001
Ex-vivo T-cell depletion	184 (44%)	58 (29%)	
CNI-based	235 (56%)	139 (71%)	
HLA			0.35
Unrelated Identical	227 (54%)	100 (51%)	
Related Identical Sibling	117 (28%)	61 (31%)	
Unrelated Non-identical	41 (10%)	20 (10%)	
Related Haploidentical	33 (8%)	14 (7%)	
Related Twin	0	2 (1%)	
Related Non-Identical	1 (<1%)	0	
Patient CMV positive	237 (57%)	114 (58%)	0.76
Donor CMV positive	200 (48%)	98 (50%)	0.56

Abbreviations: BMI, Body Mass Index; eGFR, Estimated GFR; HCT-CI, Hematopoietic Cell Transplant-specific Comorbidity Index; GVHD, Graft vs Host Disease; TCD, T-Cell Depleted Transplant; TBI, Total Body Irradiation; cGy, centiGray; HLA, Human Leukocyte Antigens; CMV, Cytomegalovirus.

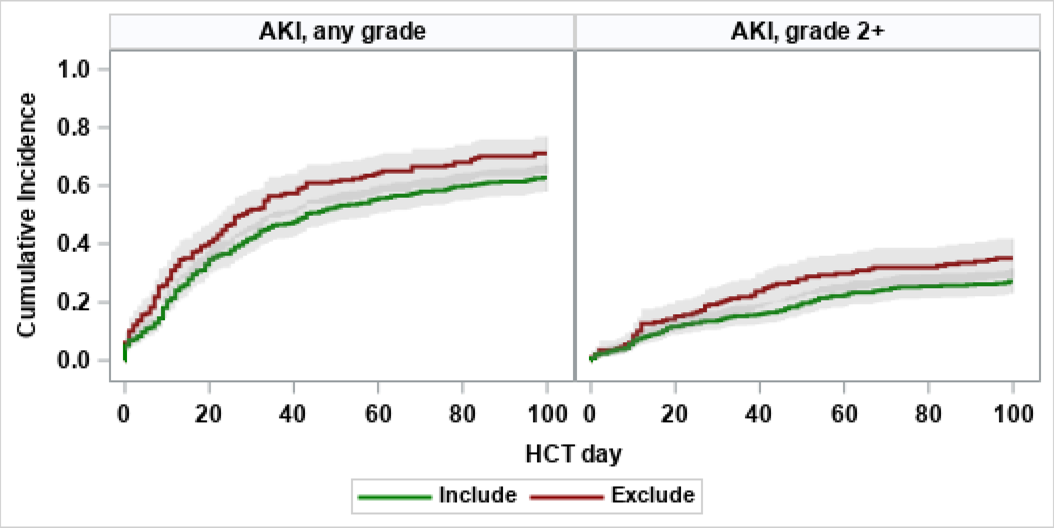

Supplemental Figure 2. Cumulative incidence of any grade AKI and grade 2+ AKI in 100 days of follow-up post-HCT, by study inclusion.

Supplemental Table 3. Results from competing-risks Cox proportional-hazards regression (adjusted analysis)

	Any grade AKI		Grade 2+ AKI	
	HR (95% CI)	P	HR (95% CI)	P
Simpson reciprocal DI				
HR for every 10-unit increase				
Baseline	0.94 (0.86, 1.03)	0.18	0.93 (0.80, 1.09)	0.38
Time-dependent covariate	1.02 (0.89, 1.16)	0.80	0.81 (0.67, 0.98)	0.02
Peri-engraftment*	1.02 (0.71, 1.48)	0.90	0.63 (0.34, 1.17)	0.14
Shannon DI				
HR for every 1-unit increase				
Baseline	0.88 (0.74, 1.06)	0.17	0.93 (0.68, 1.26)	0.62
Time-dependent covariate	1.88 (0.42, 8.5)	0.41	0.28 (0.03, 2.42)	0.25
Peri-engraftment*	1.08 (0.05, 22.6)	0.96	0.04 (0.001, 2.05)	0.11

*Landmark analysis starting at day 21 post-HCT

Table S3. Multivariate analysis of Table 2, adjusting for the following co-variates: patient ethnicity, baseline albumin, conditioning intensity, conditioning regimen and GVHD prophylaxis group.

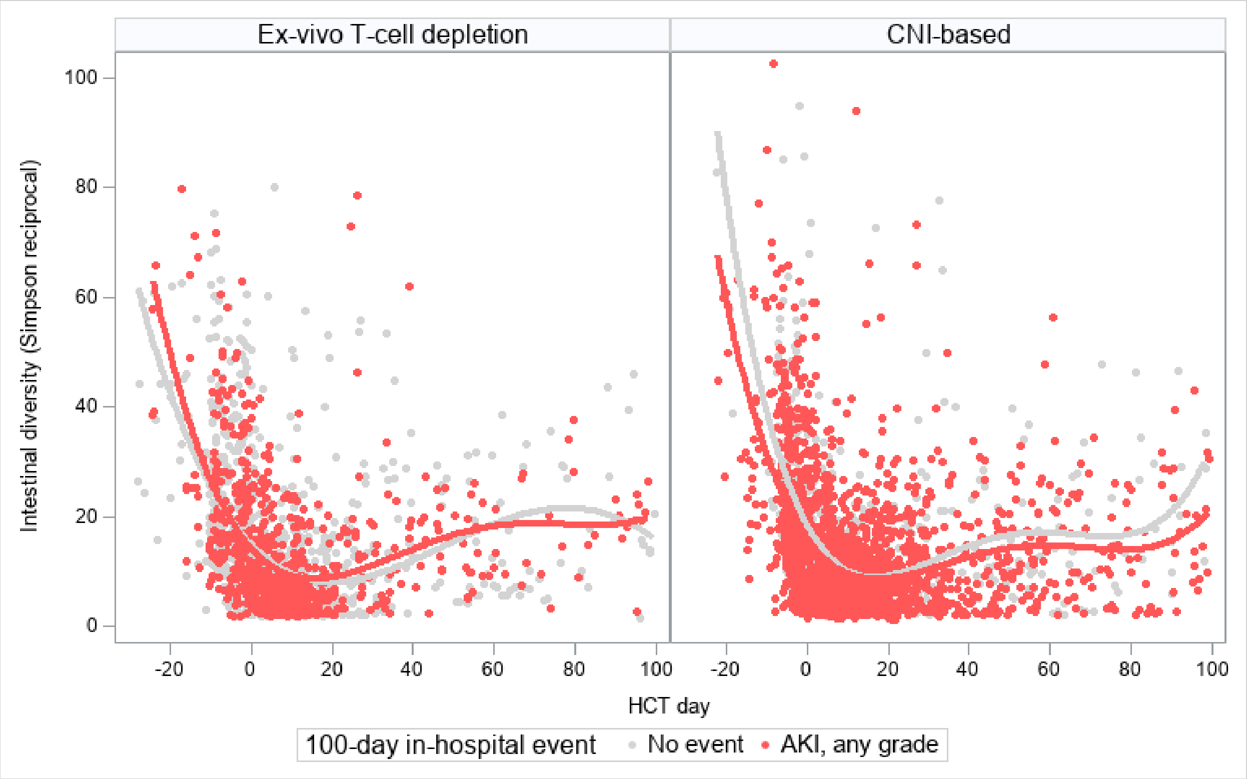

Supplemental Figure 4. Changes in Diversity Index over time comparing T-Cell Depletion and CNI-based GVHD prophylaxis, and stratified by AKI status
